# Supplementary material for: Minimum Dietary Diversity for Women of Reproductive Age (MDD-W) Data Collection: Validity of the List-Based and Open Recall Methods as Compared to Weighed Food Record
Source: Nutrients. 2020 Jul 9;12(7):2039. doi: 10.3390/nu12072039 (PMC7400839; doi:10.3390/nu12072039)
Supplement: Supplementary file 1 [file nutrients-12-02039-s001.pdf]

ONLINE SUPPORTING MATERIALS

Table S1. Associations between proportions achieving Minimum Dietary Diversity for Women of Reproductive Age from proxy methods and weighed food record proportion

	**List-based recall**† (n=1,337)			**Open recall**† (n=1,337)		
**Independent variable**	OR	SE	ICC	OR	SE	ICC
Weighed food record (n=1,337)	12.2***	2.1	0.149	13.6***	2.2	0.147

†Mixed effects logistic regression model random intercept: country. **P*<0.05, ***P*<0.01, and ****P*<0.001. OR, odds ratio; SE, standard error; ICC, intraclass coefficients.

Table S2. Food categories used during list-based recall of Minimum Dietary Diversity for Women of Reproductive Age in Cambodia, Ethiopia, and Zambia

	**Food categories**
**Required**	
A	Foods made from grains
B	White roots and tubers and plantains
C	Pulses (beans, peas and lentils)
D	Nuts and seeds
E	Milk and milk products
F	Organ meat
G	Meat and poultry
H	Fish and seafood
I	Eggs
J	Dark green leafy vegetables
K	Vitamin A-rich vegetables, roots and tubers
L	Vitamin A-rich fruits
M	Other vegetables
N	Other fruits
**Optional**	
O[†]	Insects and other small protein foods
Q	Other oils and fats
R, S, T	Savoury and fried snacks, sweets, and sugar-sweetened beverages
**Required**	
U	Condiments and seasonings
V	Other beverages and foods

[†]Only in Cambodia and Zambia, as determined by survey designers during adaptation process. Rows A–N aggregated during analysis into the 10 MDD-W food groups.

Table S3. Proportions of non-pregnant women (15-49 years) having consumed food groups and achieved Minimum Dietary Diversity for Women of Reproductive Age in Cambodia, Ethiopia, and Zambia, based on weighed food record, list-based, and open recall methods

	Cambodia			Ethiopia			Zambia		
	Weighed food record (n=430)	List-based recall (n=430)	Open recall (n=430)	Weighed food record (n=431)	List-based recall (n=431)	Open recall (n=431)	Weighed food record (n=476)	List-based recall (n=476)	Open recall (n=476)
All starchy staple foods	100	99.3	100	100	100	100	100	99.6	100
Beans and peas	11.2	16.7**	17**	80.3	90.3***	89.8***	34	34.5	34.9
Nuts and seeds	2.8	12.1***	7.4**	0.7	4.6***	3.7***	8.6	9.7	10.5
Dairy	0.7	4.7***	4.2***	4.2	7.2***	7.2***	3.8	7.8***	6.7**
Flesh foods	98.8	99.3	99.3	8.8	10.7*	10.7	39.1	51.9***	47.5***
Egg	25.1	27.9	24.9	5.8	6.5	6	34	34.7	32.4
Dark green leafy vegetables	49.5	76.1***	75.8***	6.7	11.8***	8.7*	62.6	71.4***	71.4***
Vitamin A-rich fruits and vegetables	26.7	28.1	22.8	11.6	15.1*	11.1	39.5	40.3	35.5
Other vegetables	84	84.4	84.7	94	96.5	97.2*	97.7	95.4	95.6
Other fruits	37	59.5***	39.3	4.4	10.9***	6.5	3.6	24.2***	20.6***
MDD-W	44.2	64.9***	57.2***	7.7	15.6***	11.6***	37.8	55***	48.7***

Statistically different proportions between weighed food record and proxy methods are indicated by *P<0.05, **P<0.01, and ***P<0.001.
MDD-W, minimum dietary diversity for women of reproductive age.

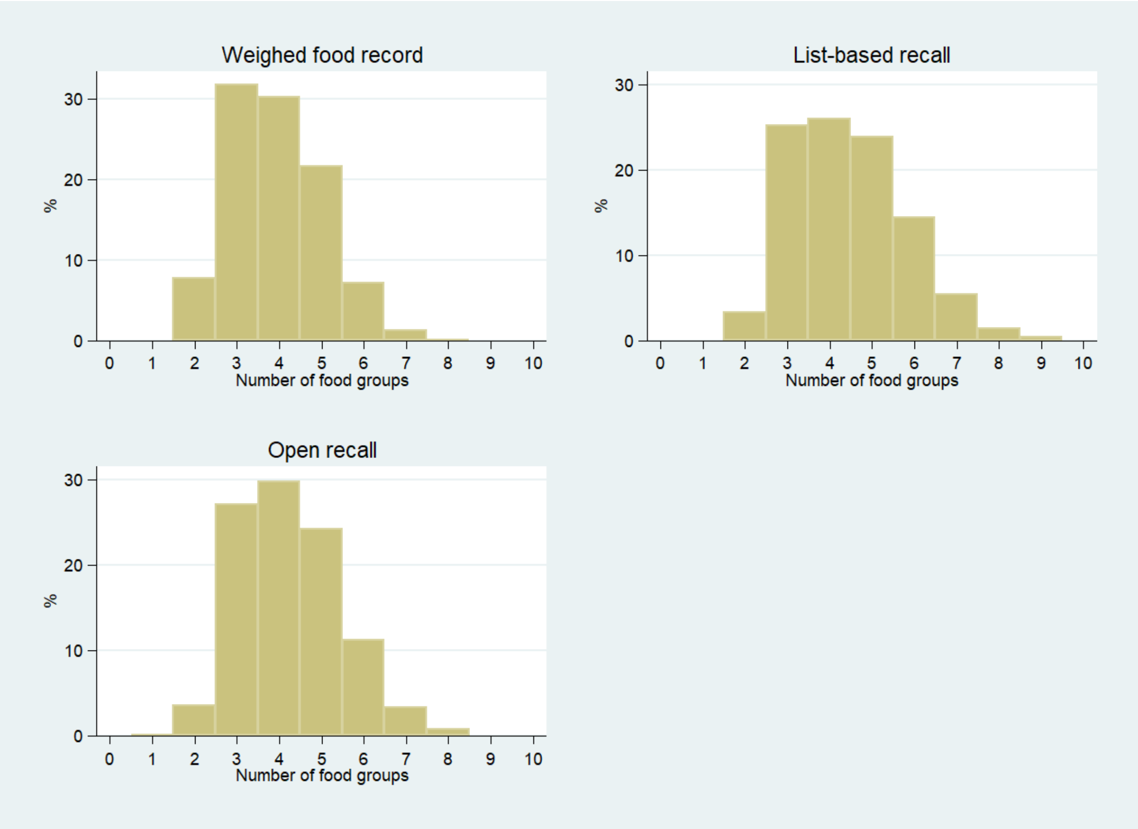

Figure S1. Distribution of ordinal food group diversity score, by dietary assessment method
